# Working together in Aboriginal health: a framework to guide health professional practice

**DOI:** 10.1186/s12913-020-05462-5

**Published:** 2020-07-01

**Authors:** Annabelle M. Wilson, Janet Kelly, Michelle Jones, Kim O’Donnell, Sandra Wilson, Emma Tonkin, Anthea Magarey

**Affiliations:** 1 College of Medicine and Public Health, Flinders University, GPO Box 2100, Adelaide, South Australia 5001 Australia; 2 Adelaide Nursing School, Adelaide University, Adelaide, South Australia 5005 Australia; 3 College of Education, Psychology and Social Work, Flinders University, GPO Box 2100, Adelaide, South Australia 5001 Australia; 4 Aboriginal Primary Health Care Unit, Murray Bridge, South Australia 5253; 5 College of Nursing and Health Sciences, Flinders University, GPO Box 2100, Adelaide, South Australia 5001 Australia

**Keywords:** Aboriginal, Indigenous, Health, Relationships, Health professional, Working together

## Abstract

**Background:**

Working effectively with Aboriginal and Torres Strait Islander people is important for maximising the effectiveness of a health care interaction between and Aboriginal and Torres Strait Islander patients and a health professional. This paper presents a framework to guide health professional practice in Aboriginal and Torres Strait Islander health.

**Methods:**

This qualitative study was based in a social constructionist epistemology and was guided by a critical social research methodology. Two methods were employed: interviews with Aboriginal health workers and allied health professionals about their experiences of working together in Aboriginal health, and an auto-ethnography conducted by the researcher, a non-Aboriginal dietitian and researcher who worked closely with two Aboriginal communities while undertaking this research.

**Results:**

Interviews were conducted with 44 allied health professionals and Aboriginal health workers in 2010. Critical Social research, which involves the deconstruction and reconstruction of data, was used to analyse data and guided the evolution of themes. Strategies that were identified as important to guide practice when working respectfully in Aboriginal health included: Aboriginal and non-Aboriginal people working with Aboriginal health workers, using appropriate processes, demonstrating commitment to building relationships, relinquishing control, having an awareness of Aboriginal history, communication, commitment, flexibility, humility, honesty, and persistence. Reciprocity and reflection/reflexivity were found to be cornerstone strategies from which many other strategies naturally followed. Strategies were grouped into three categories: approach, skills and personal attributes which led to development of the Framework.

**Conclusions:**

The approach, skills and personal attributes of health professionals are important when working in Aboriginal health. The strategies identified in each category provide a Framework for all health professionals to use when working with Aboriginal and Torres Strait Islander people*.*

## Background

Aboriginal and Torres Strait Islander peoples (from herein referred to as Aboriginal) have a strong and rich history as the oldest cultures in the world, demonstrating strength, tenacity and resilience. The advent of the Closing the Gap Campaign in Australia in 2007 [1, 2] widely publicised the unacceptable gap in life expectancy between Aboriginal and non-Aboriginal Australians, with Aboriginal men and women having a life expectancy of 8.6 and 7.8 years less than non-Aboriginal Australians respectively, and recent data shows that this gap has not narrowed over time [3]. Australian Aboriginal people also experience a significantly higher burden of chronic disease than non-Aboriginal Australians including higher rates of diabetes and kidney disease [4]. This unacceptably large gap in disease and consequently life expectancy [5] is linked to poorer social determinants of health, discriminatory practices, and political and historical marginalisation [6]. This has led to many Aboriginal people being reluctant to access health care [7] and highlights the important role that health professionals have in shaping health care interactions with Aboriginal people to be positive and directed towards the needs of the Aboriginal patient. Given this, improving Aboriginal people’s service engagement through, among other things, enhanced cultural competency of health professionals and services is an important element of addressing these inequitable health outcomes [8].

A body of literature exists that provides guidance for health professionals about how to work with Aboriginal people. Much of this is directed at non-Aboriginal health professionals and includes guidelines [9, 10], protocols [11, 12], ethical principles [13] and suggested professional practices based on research and experience [14, 15]. Authors highlight the need to work in partnership [16–20], be flexible [21], build relationships [17, 21], get to know Aboriginal people [17, 22] and reflect on their positions of privilege [23, 24]. Programs often expand on the ‘build relationships’ principle to highlight that shared trust and respect are required in order to bring about sustained health change in Aboriginal communities [17, 23, 25, 26]. However, this literature typically does not provide concrete examples of *how to action* these broad principles, particularly in relation to working together in cross-cultural teams in Aboriginal health. Thus, despite this body of evidence, unacceptable disparities in health outcomes and healthcare access between Aboriginal and non-Aboriginal Australians persist [4]. This indicates that there is still something missing in the information currently available for health professionals working in Aboriginal health.

In her address at Congress Lowitja 2012, Pat Anderson, Chairperson of the Lowitja Institute (Australia’s national institute for Aboriginal and Torres Strait Islander health research), called for a ‘deeper re-setting of the relationship between Aboriginal and non-Aboriginal Australia, and the establishment of a more equal, respectful relationship between us’ [27]. She spoke of the need for *respect for each other*, in particular a focus on ‘Closing the Gap’ in respect between Aboriginal and non-Aboriginal Australians [27]. She also identified the need for people to respect the evidence, to recognise the positive changes already underway in many communities, and for respect of the non-Aboriginal health professionals and researchers who had worked for many years alongside Aboriginal communities [27].

The purpose of this paper is to respond to the above call to action and develop a Framework for health professionals to use to guide their practice in Aboriginal health. This Framework present both *evidence* for and *examples of strategies* shared by Aboriginal and non-Aboriginal health professionals, that demonstrate *respect* when working in Aboriginal health*.* Health professionals can draw on these examples of evidence and strategies in their own practice, and use the Framework to develop an environment conducive to strong cross-cultural relationships.

## Methods

### Ethical considerations

Ethics approval for this study was received from the Flinders University Social and Behavioural Research Ethics Committee, the SA Health Human Research Ethics Committee and the ethics committees of the Aboriginal Health Council of South Australia and the Department of Education and Children’s Services. All participants provided written informed consent.

The research was guided by the National Health and Medical Research Council’s values and ethics for doing research with Aboriginal communities [28], specifically the principles of equality, respect, responsibility, reciprocity, survival and protection and spirit and integrity. These strategies were upheld throughout the research, through engagement in activities including attending events in the local communities, talking to local Aboriginal people at community lunches, working with Aboriginal project mentors, consulting with Elders’ Committees, and activities of reciprocity (giving back) based on what was requested by the community. These activities were a key strategy used to build relationships with local Aboriginal people for this research. The primary researcher (AW) worked in partnership with Aboriginal project mentors to design and undertake the project. Two of these mentors are authors on this paper (SW and KOD).

### Theoretical framework

This study used a social constructionist epistemology [29, 30], which purports that reality is experienced, or constructed, by the individual [31]. This approach avoids privileging one type of knowledge over another (for example Aboriginal or non-Aboriginal knowledge]. A critical theoretical approach was utilised to challenge oppressive structures in health care and society [32, 33] and critical social research [34] and auto-ethnography [35] were employed as methodologies. Critical social research is comprised of nine elements which provide guidance about key concepts to investigate in a research project (abstraction, essence, totality, structure, praxis, ideology, history and deconstruction and reconstruction) [34]. Autoethnography is referred to as ‘an autobiographical form of writing’ (p. 139), a ‘method and a text’ (p. 140) and a way of incorporating one’s own life experience when writing about research [35]. These methodologies were operationalised as two methods respectively, interviews with allied health professionals and Aboriginal Health Workers^1^; and an auto-ethnographic reflexive journal maintained by the researcher.

### Recruitment

Allied health professionals (HPs) and Aboriginal health workers (AHWs) working at one rural and one metropolitan community health centre in South Australia were invited to participate in an interview. The researcher was part of a healthy eating and physical activity program that was based in these two communities and this was the rationale for the selection of sites. Due to this, the researcher was known to most of the participants prior to study commencement. Purposive sampling was used to ensure that participants had relevant experiences to contribute that were information rich [36, 37], that is that participants had experiences to share about working in Aboriginal health. Recruitment was face-to-face and by email. Allied health professionals included dietitians, occupational therapists, health promotion workers and speech pathologists. Some professionals were working as allied health professionals while others had a project coordination or management role. Additional dietitians working across South Australia were also invited to participate following a presentation at a state nutrition meeting and an email flyer sent to this group. The inclusion of additional dietitians was linked to the professional background of the researcher (AW).

### Data collection

Data were collected through semi-structured interviews lasting 30 to 90 min in 2010. The interviews were conducted by AW (a female PhD Candidate with a Bachelor of Nutrition and Dietetics (Honours) and prior experience with qualitative research) at community health centres in the two communities, or at a location chosen by the participants. Interviews were based on trigger questions developed from the researchers’ experiences working with Aboriginal communities and modified following discussion with Aboriginal project mentors. The interview schedules were based around how Aboriginal and non-Aboriginal people work together in health. Probes included Aboriginal peoples’ understandings of health, beliefs about working with Aboriginal people and the impact of this on a health professionals’ practice, barriers and enablers to practice in Aboriginal health, learnings from practice and what non-Aboriginal people need to know when working with Aboriginal people. Interview schedules were developed in conjunction with Aboriginal project mentors and had between eight to 13 items (depending on cultural and professional background and position) and have been included as supplementary files. The interview schedule used with non-Aboriginal health professionals has previously been published [38, 39]. Participants were aware of the aims of the study and the interviewer’s interest in the topic. Interviews were conducted until data saturation was achieved [40].

The researcher (AW) undertook an auto-ethnography and kept a reflexive journal as part of this study; more extensive findings from this journal are published in an earlier paper [41]. The researcher kept a log of all interactions with Aboriginal and non-Aboriginal people that were relevant to the research, and notes after interviews, and summarised these in the reflexive journal when issues and challenges arose. This was an opportunity for her to reflect on her research practices and experiences and also brainstorm ways to address challenges.

### Data analysis

Interviews were audio recorded and then transcribed verbatim and deidentified through assigning each transcript the code AHW (Aboriginal Health Worker) or HP allied health professional (HP) followed by a unique number. Transcripts and the reflexive journal were imported into QSR NVivo 8.0 software (QSR International, Doncaster, Victoria, 2008). Data were coded into themes using an inductive approach [40] that recurred throughout the interview and reflexive journal by the researcher (AW). Critical social research was the methodology used for data analysis. Critical social research involves deconstruction and reconstruction of data, using ten specific questions, to guide the emergence and evolution of themes through breaking the data down into its individual elements and then putting it back together in a different way to expose deeper meaning [34]. Participants were given the option to review their transcript and check that it was accurate and that their ideas were presented appropriately.

## Results

### Characteristics of the participants

A total of 35 allied health professionals (HP) and nine Aboriginal health workers (AHW) participated in this study (44 participants). All of the allied health professionals were non-Aboriginal. Forty-one participants were female and three were male. Sixteen were from a metropolitan community (defined as major cities, Modified Monash Category 1, using the Modified Monash Model) [42] and 28 were from a rural community. More specifically, 16 were living in large rural towns (MMM3), three in medium rural towns (MMM4), eight in small rural towns (MMM5) and one in remote communities (MMM6) [42]. The length of experience of the allied health professionals working in Aboriginal health varied between 0 and 1 year (*n* = 8), 1–5 years (*n* = 13), 5–15 years (*n* = 7) and more than 15 years (*n* = 7).

**Fig. 1:**
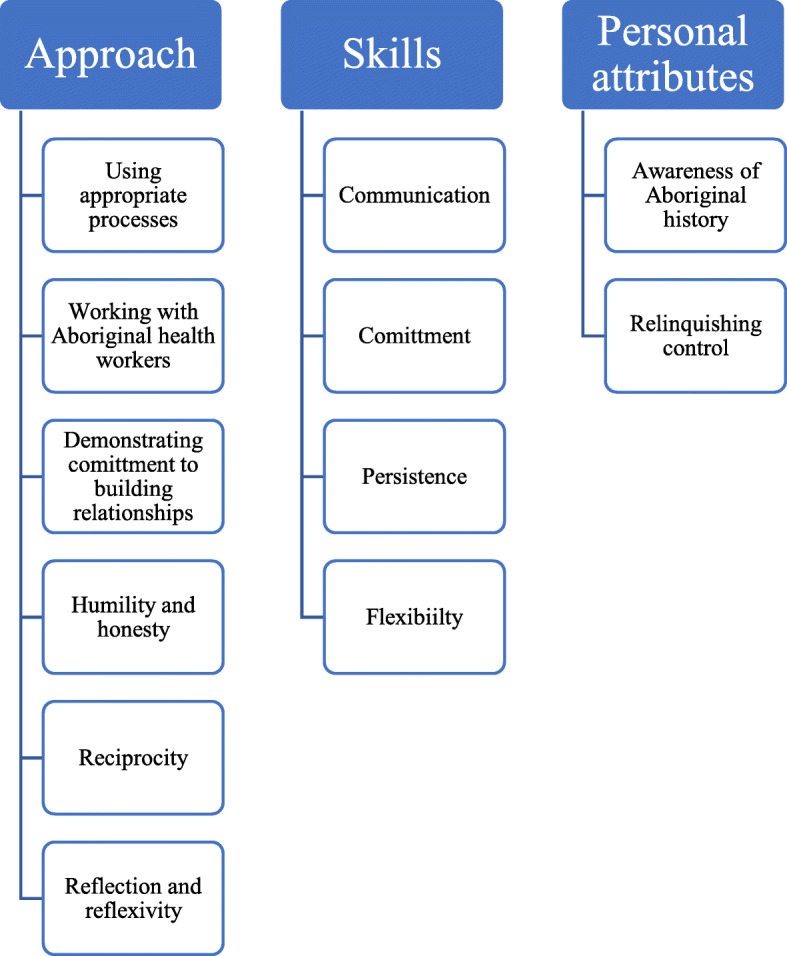
Framework of Strategies to guide practice in Aboriginal health

Participants identified and described strategies they used to work respectfully and effectively within Aboriginal health. These were further classified into three categories: approach, skills and personal attributes and are presented as a Framework (Fig. 1). These strategies also emerged as central in the auto-ethnography. Table 1 shows which types of health professionals (allied health professional versus Aboriginal Health Workers) identified which strategies as important. In this section, we describe why these strategies were important to interviewees, and then, where appropriate, how they were demonstrated by the researcher as recorded in the auto-ethnography.

**Table 1:** Usual strategies for working in Aboriginal health, and which participants identified each strategy as important

Category	Strategy	Aboriginal Health Workers	Allied health professionals
Approach	Using appropriate processes	✓	✓
	Working with Aboriginal health workers	✓	✓
	Demonstrating commitment to building relationships	✓	✓
	Humility & honesty		✓
	Reciprocity	✓	✓
	Reflection & reflexivity		✓
Skills	Communication	✓	✓
	Commitment	✓	
	Persistence		✓
	Flexibility	✓	✓
Personal attributes	Awareness of Aboriginal history	✓	✓
	Relinquishing control	✓	✓

#### Approach

##### Using appropriate processes

Aboriginal and non-Aboriginal participants identified the importance of using appropriate processes to work respectfully with Aboriginal communities. Aboriginal workers highlighted the need to *‘work with the right people’* (AHW3) and *‘go through the right channels’* (AHW6) which may be different in different locations. Two workers discussed the importance of accessing Aboriginal people through existing groups (AHW2; AHW8) which can help to facilitate processes, such as consultation with the community.

##### Working with Aboriginal Health workers

The majority of interviewees identified that it is vital to work with Aboriginal health workers when seeking to work respectfully in Aboriginal health. In this research, Aboriginal workers identified themselves as the people who know what is going on in the community (AHW2) and the right people to speak to (AHW3). Participants stated that working with AHWs helps to get things happening on the ground (HP7). This was also the case for the researcher who developed relationships with key workers in communities through attending local events. Working with key workers opened doors for the researcher who was able to attend women’s groups and get to know local women because of her relationship with local AHWs.

##### Demonstrating commitment to building relationships

Demonstrating commitment to building respectful relationships was identified as important and participants talked about ways to do this, including involvement in community events and being *‘prepared to actually do rather than just direct’* (HP28). A health professional highlighted the importance of relationships*: ‘you cannot evaluate the significance of a relationship with Aboriginal community’* (HP27). One health professional learnt through her involvement in a community event *‘that in order to build effective relationships with Aboriginal community and workers you really have to spend a lot of time building trust and being involved rather than just preaching to people about what they should do’* (HP28). Participating in a community event means that you have something to offer to the community and this is important because *‘Aboriginal people have had a lot taken from them over the years so it doesn’t hurt to be able to offer something’* (HP28).

It was acknowledged that without good, respectful relationships, very little could be achieved and that building relationships should be the first step when working with an Aboriginal community (HP27). Some of the benefits of good relationships noted by participants were the ability to get things done, the opportunity relationships provided to learn and the credibility they received from the person they had a relationship with, which then increased their chances to work well with the community.

*…if you build a relationship with those key people and they get to know you then they talk about you to the community and they will say oh yes, [name], she’s actually all right. (HP14)*

This was reflected in the researcher’s experience. She did not receive invitations to work with community groups until she had built relationships and demonstrated commitment. She began to recognise the trust that had developed between her and her Aboriginal colleagues when she was invited into a person’s home and received invitations to join women’s groups at events including the movies and a trip to the zoo.

Participants talked about how they build respectful relationships, many viewed it as an informal process and gave examples of informal ways to meet people, for example *‘taking the opportunity, if you have something that you need to give somebody, instead of sending it through the mail, if it’s not too much of an issue, just drop it off, like make yourself known but not in an assertive way’* (HP28) and *‘social conversation about what was happening on the weekend’* (HP28). Not all participants were confident to build relationships, and some talked about factors that held them back, such as a reluctance to build relationships because of the personal disclosure that was required (HP20). Others talked about that while the process might seem simple, it does take experience, confidence and time and *‘in reality it takes time to build relationships, it doesn’t happen like it happens in the text book’* (HP14). One dietitian, who had worked in a community for approximately 20 years, felt that she had gained a lot of trust over that time but was only really starting to make progress with nutrition messages in the last few years (HP35).

##### Humility and honesty

Being humble and honest were two qualities that interviewees identified as being necessary when working respectfully in Aboriginal health. Being humble was described as acknowledging that you can learn from Aboriginal people (HP28) and not assuming that you know anything that they do not know already (HP22). Part of being humble is accepting that it is okay to make mistakes (HP28; HP33) and then getting on with it again after making them (HP22). Being humble was associated with being genuine, one worker commented that

*…most Aboriginal people in my experience can very quickly tell if somebody is genuine and interested in understanding them (HP9).*

Honesty was described as being upfront about what you can achieve (HP7), being open and honest in general (HP2; HP12; HP28), being honest about not understanding things (HP9) and not making promises that you cannot keep, to ensure that you can deliver on what you say you will (HP3).

##### Reciprocity

Participating in two-way learning, or reciprocity, was seen as important to Aboriginal workers:

*…it is just constant with these sorts of things [research] and you don’t ever hear anything back, do you know what I mean? Like you think I did my part there but you don’t even get anything back. (AHW2)*

Other definitions of reciprocity provided by participants included activities where *‘…there’s learning on both sides so they give you something and then you give them something’* (HP23), a process that enables two-way, or both ways, learning, something that was mutually beneficial to both parties involved and demonstrated working in partnership. Interviewees provided examples of how they had demonstrated reciprocity in their work. One dietitian talked about how she opened up her home regularly for a community get-together when the community health centre was not seen as a safe place by the Aboriginal community (HP35). Another discussed how she had worked with an AHW because they *‘generally are quite established in their community and have a good understanding of where things are at’* and as a dietitian you can *‘support them as well in their own quest for general information’* (HP34). One dietitian talked about sharing things about herself, that she would not normally share with clients, before expecting Aboriginal people to share things about themselves (HP4). Further examples included putting on a lunch in return for Aboriginal people coming to their nutrition event (HP15), spending time at a local community event to help out even though it was not directly related to nutrition (HP8), helping with tasks such as collecting bush tucker (HP35), co-presenting at a conference with an Aboriginal Community Education Officer (HP35) and working with a community group to build up their skills in an area they have an interest so that when you leave you have left something behind that the community can use (HP11).

In her research, the researcher engaged in activities of reciprocity (for example regularly working with women’s groups around nutrition, attending local community events and talking about healthy eating and assisting with a cooking program for Aboriginal primary school children) in return for the input of Aboriginal people in the communities to her research.

##### Reflection and reflexivity

Interviewees who had worked more extensively in Aboriginal health talked about the usefulness of reflection as a strategy for good practice (HP2; HP11; HP 20; HP22; HP29; HP34). They identified that reflection enabled a review of their work practices, and sharing of information with other professionals, which made their job easier. Two dietitians (HP11 & HP34) specifically mentioned debriefing as a useful strategy. The researcher used reflexivity extensively throughout her PhD research. Reflexivity goes one step further than reflection and encourages consideration of how practice will be altered based on the reflections [35]. Through using a Reflexive Journal, and talking to Aboriginal project mentors and PhD supervisors, the researcher regularly considered her actions and reactions to situations, and how she might act and think differently in the future.

#### Skills

##### Communication

Communication was considered an important part of working with Aboriginal people, but it was stressed that communication is relevant when working with all people. Elements of communication mentioned by participants as important included non-verbal communication (for example smiling) and speaking to people, for example:

*…when someone walks in the door, you smile and say good morning and make them feel welcome because that’s what we’re here for and if you can’t do that well you shouldn’t be working here (AHW1)*

Some mentioned that Aboriginal people may have a preference for sitting and yarning under a tree (AHW2) and it was considered important to not look at your watch when doing this (AHW1). In her research, the researcher could be flexible and was able to spend time with Aboriginal people in ways they requested, and not be overly concerned about how long this might be. For example, she accompanied a women’s group on a trip to the zoo and during the bus trip was able to have casual conversations with the women about eating habits, food and nutrition.

Other approaches to communication mentioned by interviewees included: asking questions (AHW3; HP9; HP12; HP18; HP33;), using visuals (AHW5; HP12; HP35), teaching kids who pass the messages onto their families (AHW 3; AHW7), and taking time to explain why things are important:

*…I got told for years and years and years, you know we get told you need to do this and we’ve accepted that and we’ve done it but we’ve never known why or how …[ ]… years and years go by and we get told what to do all the time, what we have to do and I think we need to inform, particularly when it is non-Aboriginal people informing Aboriginal people about things, they need to know why. Why are you bringing this program to us? (AHW4)*

##### Commitment and persistence

Commitment and persistence were identified as important qualities when working respectfully in Aboriginal health. Commitment includes wanting to work with Aboriginal people (AHW7) and persisting with clients who may be difficult to contact (AHW2). One way to demonstrate commitment is to follow through with what you say and offer (AHW1). A commitment to building relationships with Aboriginal people is needed (AHW7) with an underlying understanding of the importance of relationship building in this line of work, for example *‘the biggest thing that you could do is build your relationship with them [Aboriginal people]’* (AHW1).

The researcher identified the importance of persistence through her work with the communities. In the initial phases of the research, when she was building relationships, she had to be persistent when contacting people and seeking their involvement in her work. However she also learnt not to take it personally when people declined to be involved. This was a reflection of the constant requests many Aboriginal people received for advice and input rather than a reflection on the researcher or her research (Reflexive Journal 30/11/2009, p. 102).

##### Flexibility

Flexibility was seen by interviewees as a key quality when working in Aboriginal health and it was described as having an awareness that in Aboriginal health, things don’t always happen the way they were planned in your diary, and being able to respond appropriately (AHW2). The importance of being flexible when working with Aboriginal people, but approaching the work like you would with anyone else, was highlighted:

*It is a different way of working…[ ]…[but] whatever you do for anyone else you need to do that with Aboriginal people (AHW1)*

Flexibility was also described as making your work fit the situation, or *‘framing your agenda with whoever you want to work with, the workers, the community or whatever and getting them to see how your agenda could benefit them’* (HP12). Other health professionals identified flexibility as being willing to adopt new strategies if initial plans do not work or do not suit the community (HP18), being willing to take indirect routes into the community (HP1; HP18), accepting that you may not have all of your results when you want them (HP22) and acknowledging that things happen in people’s lives that require a change of plan (HP33).

Flexibility was raised in relation to delivering healthy eating and physical activity programs:

*…even though it’s a very important part of life, being active and eating healthy, it needs to be tied into something that is seen as more important or delivered in a way that is not seen as a healthy eating and physical activity programme...[ ]…So you can cook healthy food and talk about healthy food but it’s because you’ve gone out and you’ve gone bushwalking and you’ve established that connection with land and then you’ve identified bush foods and then you’ve used things like mushrooms or wattle seed or whatever in the food that you’re preparing and then you can have the conversation about well this is healthy because, so it’s seeing the bigger context…(HP28)*

#### Personal attributes

##### Relinquishing control

Aboriginal workers reported that it is important to relinquish control when working respectfully in Aboriginal health. This entails working alongside Aboriginal staff rather than leading the process (AHW7) and not coming across as the expert (AHW7). Strategies suggested for relinquishing control included admitting what you don’t know (HP12), not expecting immediate change and understanding the limitations of your work (HP7; HP18; HP22). One worker talked about relinquishing control as a way to avoid practising in a way that reinforces colonisation (AHW8). For example, instead of telling people what to eat, provide choices based on what people are already eating, or work with a community in terms of what they value and what they want (AHW8). These were strategies used by the researcher when she worked with women’s groups in communities. In both cases, she asked what the group was interested in discussing, rather than suggesting what she thought was important. By being flexible about what was covered, she was able to be more spontaneous and respond to the groups’ needs.

##### Awareness of Aboriginal history

An understanding of Aboriginal history, as well as culture and lifestyle, was considered important when working respectfully with Aboriginal people:

*I think they (people working with Aboriginal people) just need to have an understanding of where we’re from, of where we’ve come from and you know it is not just about an Aboriginal person anyway it is about understanding what their lifestyle’s like, what their history is, and the family. (AHW4)*

The researcher became aware of the importance of Aboriginal history and its relevance to actions in the present through her conversations with Aboriginal people. For example:

*One thing that Veronica*^2^*said really shocked me – the recent events in the health service reminded her of what it was like on the Mission, where new people would come along and each time they would change the policies. I wondered whether the staff members responsible for the change at the health service had even considered that their actions could have such historical links. (Reflexive Journal 24/10/2009, pp. 96-97)*

This highlights the importance of having an understanding of Aboriginal history, from both data sources (interviews and autoethnography).

## Discussion

This paper presents a Framework to guide practice in Aboriginal health (Fig. 1). It identifies and describes specific strategies from Aboriginal health workers and allied health professionals for working well with Aboriginal people. These strategies are broken into three categories, as evidenced by the Framework (Fig. 1): approach, skills and personal attributes, highlighting the importance of these different areas when working in Aboriginal health. The strategies presented are supported by the data and they are a source of evidence and are important for all people working with Aboriginal people, not just in Aboriginal health, and provide a framework for action that promotes and enables respect for each other.

This paper supports and extends previous research identifying important approaches to practice when working in Aboriginal health. Working in partnership with Aboriginal people has been stressed by multiple authors [15–20, 22, 43–45] as has the importance of getting to know an Aboriginal person before direct questioning or “getting down to business” [17, 22], and considering a person’s context and history when working with them [18, 20, 23]. Linked to this, previous research supports the finding from this study that valuing and incorporating Aboriginal expertise is beneficial [17, 45], including through involving Indigenous peoples in the research process [46], is beneficial and relationship building is a fundamental element of working in Aboriginal health [17, 45]. The findings presented extend the current knowledge base by providing concrete examples of *how* these strategies can be implemented in practice.

Two strategies identified as central by the most experienced participants within this study were reciprocity and reflexivity, supporting previous research [17, 20, 21, 25]. Reciprocity was a crucial element of the researcher’s research and provided opportunities for her to develop and demonstrate many of the other strategies identified, for example develop trust, maintain relationships and gain a greater understanding of Aboriginal people and their concerns. Similarly reflexivity was crucial to the development of the researcher’s skills as a dietitian-researcher in Aboriginal health and, like the strategy of reciprocity, fostered her implementation of other strategies. Self-reflection enables practitioners to identify their own implicit biases, attitudes and privilege which are vital to assess when working with people from different backgrounds [41, 47]. Extending self-reflection to reflexivity supports the modification of practice to limit any negative effect these implicit biases may have on relationships and practice [48]. As such the strategies of reciprocity and reflexivity provide the critical starting point to conducting truly respectful practice with Aboriginal people, from which many of the other identified strategies naturally flow. This study provides an example of how reflexivity of the researcher, and the use of reciprocity, can be used to ensure effective engagement with Aboriginal communities. It details the way in which this was done, and discusses the result, which is a new contribution to the literature in this area of work. Other literature stresses the importance of critical reflection in Aboriginal health, for example by encouraging researchers to reflect on power and privilege [24].

There were slight differences between the strategies suggested by Aboriginal Health Workers and allied health professionals. These differences are outlined in Table 1. For example, Aboriginal Health Workers talked about the importance of commitment while other health professionals did not. This could be due to Aboriginal Health Workers’ previous experiences with health professionals who have not demonstrated ongoing commitment to a community, for example in the case of high staff-turnover which is evident amongst health professionals in remote areas of Australia [49]. The lack of identification of commitment as important by allied health professionals suggests it is important to in health professional training that this is valued by Aboriginal Health Workers with whom allied health professionals are likely to work with. Similarly, health professionals talked about the importance of reflection and reflexivity while Aboriginal Health Workers did not. This could be because health professionals in this study may have had a greater need to reflect on their practice with Aboriginal people compared to Aboriginal Health Workers who may have been more confident.

While some of the strategies reported in this paper may be unique to the Aboriginal health context (for example an understanding and awareness of colonisation), some are also transferable to other contexts. For example communication was reported as an important strategy for relationship building between health care professionals and parents of adolescents with health issues [50]. Similarly, building relationships with mothers experiencing addiction requires good communication, collaboration and cultural competency from health professionals [51]. Similarities are also evident between this work and the body of literature around working with Aboriginal communities from Canada and are therefore likely to have relevance to working with Indigenous people globally. For example, having a community presence was identified as important in two studies [52, 53], which is similar to the strategy ‘reciprocity’ from this study, which involves being present in the community. Using appropriate processes, or identifying community protocols, is also reported elsewhere [52].

A strength of this research is that it considered the views of both Aboriginal and non-Aboriginal people, and by using a social constructionist epistemology, did not privilege one type of knowledge over the other. A limitation is that this study did not include practitioners working in remote areas, who often work with Aboriginal people in Australia. The data is also 10 years old (collected in 2010), While there have been contextual changes in health settings in South Australia since then (for example closure of some community health centres due to changed Government funding), the issues and challenges around working in Aboriginal health still exist and are discussed in current literature [18] and can be applied to other settings including acute health care services [54].

## Conclusions

This paper presents a framework of strategies for how health professionals can work effectively with Aboriginal people. It describes *how* Aboriginal Health Workers and allied health professionals implemented key strategies in their practice, a description that is lacking in the literature. Health professionals will benefit from embracing these strategies when working in Aboriginal health to maximise working respectfully with Aboriginal people. It highlights that through commitment to the strategies identified in this paper Aboriginal and non-Aboriginal people can practice *respectfully* in Aboriginal health*.* Further research could address how Aboriginal and non-Aboriginal health professionals could be supported to implement the strategies presented in this paper in their work and how demonstrating respect, using these strategies, may contribute to a reduction in the inequitable gap in health and life expectancy between Aboriginal and non-Aboriginal people in Australia.

## Supplementary information

**Additional file 1.** Interview Schedules.

## Data Availability

The datasets generated during and/or analysed during the study are not publicly available due to ethical restrictions but may be available from the corresponding author on reasonable request.

## Abbreviations

AHW: Aboriginal Health Worker
HP: Allied Health Professional

## Footnotes

1 Aboriginal Health Workers are Aboriginal and/ or Torres Strait Islander people who have undertaken a minimum of Certificate 111 in Aboriginal and Torres Strait Islander Health Care and may have undertaken further specialised training

2 Name has been changed to maintain confidentiality
