# Supplementary material for: Working together in Aboriginal health: a framework to guide health professional practice
Source: BMC Health Serv Res. 2020 Jul 1;20:601. doi: 10.1186/s12913-020-05462-5 (PMC7329497; doi:10.1186/s12913-020-05462-5)
Supplement: Supplementary file 1 — Additional file 1. Interview Schedules. [file 12913_2020_5462_MOESM1_ESM.docx]

**Interview Schedules**

**Non-Aboriginal participants (previously published in Wilson et al 2015 and Wilson et al 2016)**

1. I would like to talk now about your role as …… at …….., and any experiences you have had

with the Aboriginal community through that role.

1. What do you see your role, as …………, to entail in terms of working with the Aboriginal community?
2. During your time as ……….., did you attempt to work with the Aboriginal community? If no – why not? If yes – explore: process of engagement/ contact, projects worked on, outcomes and barriers/ enablers
3. What learnings have come out of your work with the Aboriginal community?
4. What do non-Aboriginal people need to know when working with Aboriginal people?
5. How do you demonstrate a commitment to Aboriginal health through your work?
6. As a non-Aboriginal person working ……….., what stops you or helps you to work with the Aboriginal community?
7. What are some of the beliefs that non-Aboriginal people hold about working with Aboriginal communities? How do you think that this impacts on their work?
8. Do you think that colonisation still impacts on the lives of Aboriginal people? Would you address this in the way that you work with Aboriginal people?
9. Do you have any other comments or is there something you thought I would ask that I have not?
10. In your experience are new graduate health professionals equipped to work with Aboriginal people? Why or why not?

*References*

*Wilson, A., Magarey, A. M., Jones, M., O'Donnell, K. M., & Kelly, J. (2015). Attitudes and characteristics of health professionals working in Aboriginal health. Rural and Remote Health, 15: 2739.*

*Wilson, A. M., Kelly, J., Magarey, A., Jones, M., & Mackean, T. (2016). Working at the interface in Aboriginal and Torres Strait Islander health: focussing on the individual health professional and their organisation as a means to address health equity. International Journal for Equity in Health, 15(1), 187.*

**Aboriginal participants**

1. I would like to talk to you about your role as …… at …… and any experiences you have had through that role. In relation to that role, how have (local programs/ practitioners within your health service) worked with Aboriginal people in your community?
2. What do you think makes an Aboriginal person healthy? Is this different to what makes a non-Aboriginal person healthy? How is it the same or different? Why?
3. How have (local programs/ practitioners within your health service) supported you/ your organisation in your work? Is there anything else that could have been offered that would have been useful for yourself and/ or the Aboriginal community?
4. How have (local programs/ practitioners within your health service) had an impact on the lives of Aboriginal people in the local community?
5. How do staff at the local health service engage with you? What processes do they use? Were these suitable (Why or why not?)
6. What things should non-Aboriginal staff know about working with Aboriginal staff and/ or the Aboriginal community?
7. How can (local programs/ practitioners within your health service) best work with (a) Aboriginal staff members and (b) with Aboriginal community members?
8. Do you have any other comments or is there something you thought I would ask that I have not?
